# Correspondence

**Published:** 1861-04

**Authors:** E. L. Connally

**Affiliations:** Naval Hospital, Warrington, Fla.


[CORRESPONDENCE OF THE JOURNAL.]

Naval Hospital,

Warrington, Fla., March 25th, 1861.

Dear Sir:—It is with pleasure, I take the opportunity of placing at your disposal a list of the diseases treated in the Hospital at this place, but regret the imperfection of this notice of diseases in the Army, particularly as regards the treatment. This is, however, unavoidable in an irregular report from observations made in so short a stay at this place. Dr. H. M. Hunter, of Eufaula, Ala., Surgeon of 1st Regiment Alabama troops, has charge of the Hospital here. He is a sociable, genteel kind of a man, and is in every way worthy of the position he occupies. His assistant, Dr. Warren, of Clayton, Ala., is an excellent man, and bids fair to be one of the brightest lights in the profession.

The following cases have been under treatment in the Hospital, from the 16th of February to the 25th of March:

*Ophthalimia*, 2 Cases—Treatment—cold water to eyes, and general sedatives—Cups to temple, in one case—recovery in 4 and 10 days respectively.

*Concussion* of spinal marrow, producing partial paralysis of the bladder and lower extremeties—*treatment*, stimulants, and exciting liniments over lumbar spine—discharged, greatly improved, in 23 days.

*Tympanitis*—under treatment 6 days—recovery.

*Cholera Morbus*, " 2 " "

*Constipation*, " 5 " "

*Intermittent Fever*, 4 cases average of 9½ "

*Typhoid Fever*, " 31 " "

*Neuralgia*, " 1 " "

*Diarrhœa*, 2 cases, " 7 and 2 " "

*Convulsions*, " 3 " "

*Catarrh*, 7 cases, " average of 4½ " "

*Pulmonary affection*, 18 " "

*Ulcer of the leg*, " 10 " "

*Stricture of the Urethra* 4 " "

*Cephalalgia*, " 3 " "

*Tonsilitis*, 2 cases, " 3 " "

*Contusion of Scalp*, " 14 " convalescent.

*Fractured Clavicle with pleuro-pneumonia*, " 10 " "

*Gunshot wound of penis and hip*, " 7 " improving.

*Dysentery, chronic*, 2 cases, 5 " convalescent.

*Delirium Tremens* " 3 " improving.

*Gonorrhœa*, 8 cases, " average 6 " 2 convales'nt, 6 cured.

*Syphilis, primary*, " with mercury, 16 " recovery.

*Syphilis Secondary*, 2 cases 4 and 16, improving.

These were treated with mercurials—one of them, with a fungous growth in the nose, was subjected to canterization. I witnessed to-day the expertness of the Surgeon and Assistant with surgical instruments. In them it was exhibited in a degree characteristic of good surgeons. The operation was amputation of the arm, four inches above the elbow, on account of the accidental discharge of a musket, while in drill, the load, (ball and three shot), passing up through the fore arm in such a manner as to make amputation inevitable. The patient being placed under the influence of chloroform and morphia, the arm was amputated by a circular cut, and the skin and edges of the cut surface drawn together over the end of the bone, with isinglass plaster. He is now (four hours after the operation) comfortable, and pulse normal.

We leave to-morrow morning for Fort McRae, where we will be permanently located. From there you will hear from me, when anything of interest in Medicine or Surgery presents itself. Truly,

E. L. CONNALLY, M. D.

